# Using Structural Equation Modeling to Untangle Pathways of Risk Factors Associated with Incident Type 2 Diabetes: the Lifelines Cohort Study

**DOI:** 10.1007/s11121-022-01357-5

**Published:** 2022-03-01

**Authors:** Ming-Jie Duan, Louise H. Dekker, Juan-Jesus Carrero, Gerjan Navis

**Affiliations:** 1 Department of Internal Medicine, University Medical Center Groningen, Hanzeplein 1, P.O. Box 30 001, 9700RB Groningen, The Netherlands; 2 National Institute for Public Health and the Environment, Bilthoven, The Netherlands; 3 Department of Medical Epidemiology and Biostatistics, Karolinska Institutet, Stockholm, Sweden

**Keywords:** Conceptual model, Path analysis, Risk factors, Structural equation modeling, Type 2 diabetes

## Abstract

Risk factors for type 2 diabetes are multifaceted and interrelated. Unraveling the complex pathways of modifiable risk factors related to incident type 2 diabetes will help prioritize prevention targets. The current analysis extended a previously proposed conceptual model by Bardenheier et al. (*Diabetes Care*, 36(9), 2655–2662, 2013) on prediabetes with a cross-sectional design. The model described the pathways of four aspects of modifiable risk factors in relation to incident type 2 diabetes, including socioeconomic status (income and education); lifestyle behaviors (diet quality, physical activity, TV watching, smoking, risk drinking, and unhealthy sleep duration); clinical markers (HDL-cholesterol, triglycerides, BMI, and waist circumference); and blood pressure. We performed structural equation modeling to test this conceptual model using a prospective population-based sample of 68,649 participants (35–80 years) from the Lifelines cohort study. During a median follow-up of 41 months, 1124 new cases of type 2 diabetes were identified (incidence 1.6%). The best-fitting model indicated that among all modifiable risk factors included, waist circumference had the biggest direct effect on type 2 diabetes (standardized *β*-coefficient 0.214), followed by HDL-cholesterol (standardized *β*-coefficient − 0.134). Less TV watching and more physical activity were found to play an important role in improving clinical markers that were directly associated with type 2 diabetes. Education had the biggest positive effects on all lifestyle behaviors except for unhealthy sleep duration. Our analysis provides evidence to support that structural equation modeling enables a holistic assessment of the interplay of type 2 diabetes risk factors, which not only allows the estimation of their total effects but also prioritization of prevention targets. Regarding the current guideline for diabetes prevention, waist management in addition to BMI control (clinical level), as well as less TV watching in addition to more physical activity (behavioral level), may provide additional public health benefits. Better education would be the main societal goal for the prevention of type 2 diabetes.

**Supplementary Information:**

The online version contains supplementary material available at 10.1007/s11121-022-01357-5.

## Introduction

The development of type 2 diabetes is multifactorial. Besides inherited traits and age, various modifiable risk factors have been identified. Among clinical risk factors, obesity has been found to be one of the strongest risk factors for type 2 diabetes. It has been suggested that excess body fat, especially visceral fat, is central to the pathogenesis of insulin resistance (Lee et al., 2018; Neeland et al., 2019). Prospective cohort studies also found abnormal blood lipid profile, such as low HDL-cholesterol and high triglycerides, to be a strong predictor for the development of type 2 diabetes (Després & Lemieux, 2006; Kruit et al., 2010; von Eckardstein & Widmann, 2014). For lifestyle behaviors, both interventions and observational studies have demonstrated that poor diet (Maghsoudi et al., 2016; Schulze et al., 2005), physical inactivity (Astrup, 2001; Aune et al., 2015), and smoking (Pan et al., 2015) may contribute to the risk of type 2 diabetes independent of weight change. Observational studies have also established that risk drinking is associated with high risk of type 2 diabetes (Knott et al., 2015). In addition, emerging lifestyle risk factors, such as excessive TV watching (Llavero-Valero et al., 2021; Patterson et al., 2018) and unhealthy sleep duration (Cappuccio et al., 2010), have potential as new type 2 diabetes prevention targets. After controlling for the aforementioned risk factors, socioeconomic status, such as low education and insufficient income, has been found to be associated with higher risk of type 2 diabetes (Foster et al., 2018; Maty et al., 2005; Vinke et al., 2020). We present a more extensive summary of evidence in Supplementary Table 1.

In diabetes research, conventional approaches for risk identification often apply traditional regression models, in which the net effects of risk factors are estimated under the assumption of an independent direct effect on diabetes status. However, some risk factors may act as mediators (e.g., obesity, blood lipids) or mainly exert indirect effects (e.g., education, income) (Bardenheier et al., 2013; Roman-Urrestarazu et al., 2016). The lack of insight into their holistic interrelationships has led to the fragmentation of evidence and development of unfocused prevention programs. More specifically, obesity and abnormal blood lipids are largely attributed to unhealthy lifestyle behaviors, whereas all are strongly influenced by socioeconomic status. These factors, in turn, collectively form several hypothesized intersecting pathways that lead to the eventual development of type 2 diabetes (Duan et al., 2021; Foster et al., 2018; Maty et al., 2005; Vinke et al., 2020; Zhu et al., 2021). Socioeconomic status is thus considered the overarching upstream determinant of type 2 diabetes for its significant effects on proximal (or downstream) risk factors. Likewise, lifestyle behaviors are the upstream determinants of clinical disorders such as obesity (Lakerveld & Mackenbach, 2017). In terms of primary prevention, it would be highly useful to understand the relatedness of a broad range of risk factors, so that aiming at prioritized risk factor targets and their most influential upstream determinants would optimize the effectiveness of diabetes prevention at population level.

**Fig. 1:**
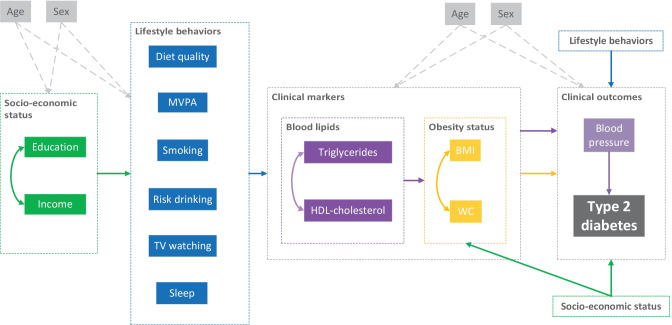
Conceptual model illustrating pathways of risk factors to incident type 2 diabetes. MVPA denotes non-occupational moderate-to-vigorous physical activity; WC denotes waist circumference; and sleep denotes unhealthy sleep duration (versus healthy sleep duration). Straight line with one arrowhead denotes a direct effect (e.g., income to MVPA), and curved line with double arrowheads denotes a correlation term (e.g., triglycerides and HDL-cholesterol). For easy reading, several factors are repeated at different locations with different pathways depicted, but they do not differ from their identical others (e.g., education and income [socioeconomic status])

To this purpose, we aimed to analyze a conceptual model (originally proposed by Bardenheier et al. on prevalent prediabetes (Bardenheier et al., 2013; Roman-Urrestarazu et al., 2016)), including multiple modifiable risk factors and their interrelationships for type 2 diabetes (Fig. 1). We extended the original conceptual model with 4 important lifestyle behaviors, i.e., TV watching (Llavero-Valero et al., 2021; Patterson et al., 2018), smoking (Pan et al., 2015), sleep duration (Cappuccio et al., 2010), and risk drinking (Knott et al., 2015). We examined this model by structural equation modeling (SEM) using data from the Lifelines cohort study, focusing on incident type 2 diabetes as outcome. SEM is a multivariate statistical technique that allows the quantification of multiple intersecting pathways (yielding path coefficients) within a conceptual model simultaneously. Untangling the pathways of these risk factors may provide the additional evidence needed to develop better prevention strategies by identifying the most crucial pathways as priority prevention targets.

## Methods

### Study Design of the Lifelines Cohort Study

The Lifelines study is a multi-disciplinary prospective general population-based cohort study that applies in a unique three-generation design to study the health and health-related behaviors of 167,729 people living in the north of The Netherlands. The Lifelines cohort study was established from year 2006 to 2013. Detailed information regarding recruitment strategy and the representativeness of the Lifelines study population are shown in Supplementary Text 1 (Klijs et al., 2015; Scholtens et al., 2015).

Four assessment rounds have taken place: T1-baseline assessment (year 2007 to 2014) and three follow-ups, i.e., T2, T3, and T4. Comprehensive physical examinations, biobanking, and questionnaires were conducted at T1 and T4 (Supplementary Fig. 1). The Lifelines study was conducted according to the principles of the Declaration of Helsinki and was approved by the medical ethical committee of the University Medical Center Groningen, The Netherlands (approval number 2007/152). All participants gave written informed consent to participate the study.

### Study Population and Exclusion Criteria

In this study, participants between the ages of 35 and 80 years who were free of diabetes at baseline from the Lifelines cohort study were included. We further excluded participants if (1) they were diagnosed with cancer or renal failure before enrollment; (2) they were pregnant at baseline; (3) they developed type 1 diabetes or gestational diabetes during follow-ups; (4) they had no available follow-up data; and (5) they had unreliable dietary intake data. Dietary intake data was considered unreliable when the ratio between reported energy intake and basal metabolic rate, calculated with the Schofield equation (Schofield, 1985), was below 0.50 or above 2.75, based on the considerations of Goldberg (Black, 2000). Furthermore, except for physical activity and income, participants with missing data on other variables (missing less than 1%) were excluded. This led to an additional exclusion of 1.7% of the study population. In this study, multiple imputation was used to deal with missing data (Kline, 2015). This additional exclusion aimed to avoid massive imputation and was not expected to have major impacts on our results. After applying exclusion criteria, in total 68,649 participants (40,121 women and 28,528 men) were included in the analysis. Supplementary Fig. 2 shows the study flow chart.

### Clinical Measurements

Blood samples were collected by venipuncture in a fasting state between 8 and 10 am. Serum levels of glucose, HbA_1c_, HDL-cholesterol, and triglycerides were subsequently analyzed. Baseline measurements of blood pressure and anthropometry were made by trained research staff following standardized protocols. Anthropometric measurements were performed without shoes and heavy clothing. Participants were considered having hypertension at baseline if they (1) used hypertensive medication (ATC codes C02, C03, C07, C08, and C09) (WHO Collaborating Centre for Drug Statistics Methodology & Norwegian Institute of Public Health, 2020); (2) had systolic blood pressure ≥ 140 mmHg; or (3) had diastolic blood pressure ≥ 90 mmHg (Williams et al., 2018). Detailed information for clinical measurements is available in Supplementary Text 2.

### Assessment of Lifestyle and Socioeconomic Covariates

Age, education level, income level, smoking status, sleep duration, TV watching time, and physical activity level were assessed by self-administered questionnaires. Age at baseline was calculated from date of birth in the questionnaire. Highest education level achieved was categorized according to the International Standard Classification of Education (ISCED): (1) low—level 0, 1, or 2; (2) middle—level 3 or 4; and (3) high—level 5 or 6 (UNESCO, 1997). Income was based on monthly household net income and was categorized as < 1000, 1000–2000, 2000–3000, and > 3000 euro/month. Smoking status was categorized as never, former, and current smoker. Unhealthy sleep duration was defined as sleep time less than 6 or more than 9 h per day (Cappuccio et al., 2010). Average TV watching time per day was asked in hours plus minutes. Physical activity level was assessed by the validated Short QUestionnaire to ASsess Health-enhancing physical activity (SQUASH) (Wendel-Vos et al., 2003), from which non-occupational moderate-to-vigorous physical activity (MVPA), including commuting and sports (both if ≥ 4.0 MET), was calculated in minutes per week, and was further divided into sex-specific quartiles (if not zero) or coded to zero (Byambasukh et al., 2020; Wendel-Vos et al., 2003).

Dietary intake was assessed using a semi-quantitative self-administered food frequency questionnaire (FFQ), which was aimed to assess the habitual intake of 110 food items (including alcohol) during the last month and was designed based on the validated Dutch FFQ (Streppel et al., 2013). The questionnaire assessed the frequency of consumption and portion sizes. The latter was estimated using fixed portion sizes (e.g., slices of bread, pieces of fruit) and commonly used household measures (e.g., cups, spoons). The food-based Lifelines Diet Score (LLDS) was calculated to evaluate the diet quality of each participant. More specifically, this score ranks the relative intake of nine food groups with positive health effects (vegetables, fruit, whole grain products, legumes/nuts, fish, oils/soft margarines, unsweetened dairy, coffee, and tea) and three food groups with negative health effects (red/processed meat, butter/hard margarines, and sugar-sweetened beverages). The development of this score is described in detail elsewhere (Vinke et al., 2018). Risk drinking was defined as consuming more than 15 g of alcohol per day, which was approximated to one drink per day.

### Ascertainment of Incident Type 2 Diabetes

Incident type 2 diabetes was assessed by self-report questionnaires (T2, T3, and T4) and blood test (T4). Participants were considered an incident case if they met either of the following criteria: (1) self-reported newly developed type 2 diabetes from last available questionnaire; (2) had fasting glucose ≥ 7.0 mmol/L; or (3) had HbA_1c_ ≥ 48 mmol/mol (6.5%) (American Diabetes Association, 2020).

### The Conceptual Model

Figure 1 illustrates the conceptual model that connects modifiable risk factors with incident type 2 diabetes and with each other, in which they are grouped into four different levels, i.e., socioeconomic status (education and income), lifestyle behaviors (diet quality [LLDS], non-occupational MVPA, smoking status, TV watching time, unhealthy sleep duration, and risk drinking), clinical markers (triglycerides, HDL-cholesterol, BMI, and waist circumference), and clinical outcomes (blood pressure and incident type 2 diabetes).

The original conceptual model was first proposed by Bardenheier et al. on prevalent prediabetes (Bardenheier et al., 2013; Roman-Urrestarazu et al., 2016). We extended the original model by adding four modifiable lifestyle behaviors (smoking, TV watching, risk drinking, and unhealthy sleep duration) and adapting several pathways based on previous evidence (Supplementary Table 1). Specifically, we hypothesized that (Fig. 1) (1) socioeconomic status had direct effects on lifestyle behaviors; (2) lifestyle behaviors had direct effects on clinical markers; (3) blood lipids (HDL-cholesterol and triglycerides) had direct effects on obesity status (BMI and waist circumference); (4) blood pressure had direct effect on incident type 2 diabetes; and (5) clinical markers had direct effects on clinical outcomes. In the conceptual model, we also allowed direct effects from socioeconomic status and lifestyle behaviors on obesity status and clinical outcomes, because there might be unobserved mediators along the causal pathways. Furthermore, age and sex, as two strong unmodifiable risk factors for type 2 diabetes, were also included in the conceptual model and were hypothesized to have direct effects on all other factors. In total, the conceptual model yielded 96 hypothesized paths and 3 correlations between the measurement errors of variables.

### Statistical Analysis

We used structural equation modeling (SEM) to examine our conceptual model (Fig. 1). SEM analysis is chiefly a confirmatory statistical technique to test if the hypothesized model is correctly specified and supported by the data observed, rather than generating new hypothesis (Kline, 2015). Because the hypothesized model consisted of ordered categorical variables (e.g., income), we used the estimation method—weighted least square with mean and variance adjustment (Muthén et al., 1997). The WLSMV is suggested to be the most suitable estimator in SEM if the model tested contains multiple binary or ordered endogenous categorical variables (Muthén et al., 1997). Additionally, we estimated the associations between each included risk factor and incident type 2 diabetes using logistic regression model as a conventional approach for risk identification.

In order to improve and evaluate model fit, the following aspects were considered. First, we referred to the model fit indices calculated from the SEM output, i.e., comparative fit index (CFI), standardized root mean square residual (SRMR), root mean square error of approximation (RMSEA), and Tucker-Lewis index (TLI). We did not purely rely on the commonly used cut-offs of these fit indices as the absolute criteria (Xia & Yang, 2019). Additionally, we performed sensitivity analyses using other estimators to cross-check the model fit. Second, modification indices, which are based on chi-square statistics indicating the changes in model’s goodness-of-fit if an omitted path was added, were also used as reference for adjustments of particular paths (Kline, 2015).

Missing data for income (proportion of missing 15.3%) and non-occupational MVPA (proportion of missing 6.4%) were imputed with chained equation creating 25 imputed datasets (Van Buuren et al., 1999), from which results were pooled according to the Rubin’s rule (Li et al., 1991).

In order to ensure the robustness of our results, we performed several sensitivity analyses. Detailed methods and results are discussed in Supplementary Text 3.

We used STATA (version 13.1) for data management and descriptive data analyses, and R Studio (version 1.1.383) with lavaan package (version 0.6–5; Y. Rosseel) for SEM analysis (Rosseel, 2012). Multiple imputation was performed with mice package (version 3.8.0; S. van Buuren et al.) in R Studio (Van Buuren & Groothuis-Oudshoorn, 2010), and results from imputed datasets were pooled with semTools package (version 0.5–2; T.D. Jorgensen et al.) in R Studio (Jorgensen et al., 2019). Statistical significance was considered if *p* value < 0.05.

## Results

### Descriptive Statistics

Among 68,649 participants (aged 35–80 years) included in the analysis, we identified 1124 type 2 diabetes cases (incidence 1.6%) after a median follow-up of 41 months. Compared with participants who did not develop type 2 diabetes throughout the study, those who developed type 2 diabetes tended to be older and male, have less education and lower income at baseline, engage in negative lifestyle behaviors, and have poorer clinical markers (Table 1).

**Table 1:** Baseline characteristics by diabetes status

**Characteristics**	**Total (*n* = 68,649)**	**Type 2 diabetes (*n* = 1124)**	**Non-diabetes (*n* = 67,525)**
Age, years	49.7 ± 9.5	54.8 ± 10.0	49.6 ± 9.4
Sex, %			
Women	58.4	49.1	58.6
Men	41.6	50.1	41.4
Fasting glucose, mmol/L	4.97 ± 0.50	5.81 ± 0.65	4.95 ± 0.48
HbA_1c_, mmol/mol	37.31 ± 3.27	41.55 ± 3.49	37.24 ± 3.22
HbA_1c_, %	5.55 ± 0.30	5.94 ± 0.32	5.55 ± 0.29
Triglycerides, mmol/L	1.19 ± 0.80	1.77 ± 1.54	1.18 ± 0.78
HDL-cholesterol, mmol/L	1.53 ± 0.41	1.30 ± 0.37	1.53 ± 0.41
BMI, kg/m^2^	26.2 ± 4.0	29.6 ± 4.7	26.1 ± 4.0
Underweight (< 18.5), %	0.4	0.1	0.4
Normal (18.5–24.9)	41.5	13.4	41.9
Overweight (25.0–29.9), %	43.0	45.6	43.0
Obese (> 30.0), %	15.1	40.8	14.7
Waist circumference, cm	91.0 ± 11.7	101.5 ± 12.1	90.8 ± 11.6
Large waist circumference^a^, %	34.2	66.6	33.6
Hypertension, %	28.8	59.4	28.3
Systolic blood pressure, mmHg	126.4 ± 15.5	134.7 ± 16.0	126.3 ± 15.4
Diastolic blood pressure, mmHg	74.9 ± 9.4	77.8 ± 10.0	74.8 ± 9.4
Lowest tertile of Lifelines Diet Score, %	28.6	32.1	28.6
Alcohol intake, g/day	4.57 (0.89, 11.11)	3.79 (0.52, 12.25)	4.64 (0.89, 11.09)
Risk drinking (> 15 g/day), %	16.7	20.3	16.6
Non-occupational MVPA, minutes/week^b^	190 (65, 370)	160 (30, 360)	190 (70, 370)
Smoking status, %			
Never	44.6	33.7	44.8
Former	38.5	46.7	38.3
Current	16.9	19.6	16.8
TV watching time, h/day	2.5 ± 1.3	3.0 ± 1.5	2.5 ± 1.3
Sleep duration, h/day	7.42 ± 0.85	7.42 ± 0.96	7.42 ± 0.85
Having unhealthy sleep duration (< 6 or > 9 h/day), %	2.97	5.42	2.93
Education, %			
Low	31.2	46.9	30.9
Middle	38.7	33.1	38.7
High	30.2	20.0	30.4
Income (euro/month), %^c^			
< 1000	3.0	5.0	3.0
1000–2000	18.5	26.2	18.3
2000–3000	30.2	30.3	30.2
> 3000	33.0	24.0	33.2

Data are expressed as unadjusted mean ± standard deviation for age, fasting glucose, HbA_1c_, triglycerides, HDL-cholesterol, BMI, waist circumference, systolic blood pressure, diastolic blood pressure, TV watching time, and sleep duration; data are expressed as median (interquartile) for non-occupational MVPA and alcohol intake; data are expressed as observed percentage for sex, obesity status, large waist circumference, hypertension, lowest tertile of Lifelines Diet Score, risk drinking, smoking status, having unhealthy sleep duration, education, and income

^a^Large waist circumference is defined as waist circumference > 102 cm (40 in.) in men and > 88 cm (35 in.) in women

^b^Non-occupational MVPA denotes non-occupational moderate-to-vigorous physical activity level. The percentages of missing data were: total 6.4%, type 2 diabetes cases 8.8%, and non-diabetes cases 6.4%

^c^For income level, the percentages of missing data were: total 15.3%, type 2 diabetes cases 14.6%, and non-diabetes cases 15.3%

### Structural Equation Model

**Fig. 2:**
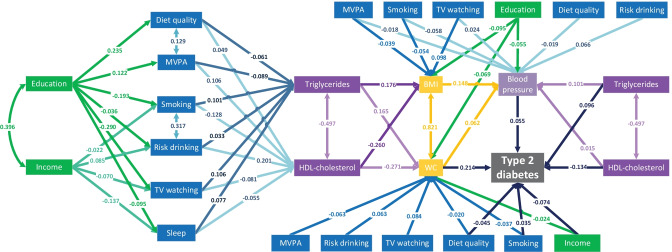
Quantified best-fit conceptual model illustrating pathways of risk factors to incident type 2 diabetes. MVPA denotes non-occupational moderate-to-vigorous physical activity; WC denotes waist circumference; and sleep denotes unhealthy sleep duration (versus healthy sleep duration). Straight line with one arrowhead denotes a direct effect (e.g., income to MVPA), and straight or curved line with double arrowheads denotes a correlation term (e.g., triglycerides and HDL-cholesterol). For easy reading, several factors are repeated at different locations with different pathways depicted, but they do not differ from their identical others (e.g., education and income [socioeconomic status]). Sample size tested for the conceptual model, *n* = 68,649. Tests for significance: *p* value < 0.001 for all path coefficients except for HDL-cholesterol to blood pressure (*p* value = 0.002) and smoking to incident type 2 diabetes (*p* value = 0.012). Adjusted for sex and age

The best-fit model (Fig. 2; CFI 0.981, TLI 0.949, RMSEA 0.032, SRMR 0.023) was achieved after we made adjustments to our original hypothesized model (Fig. 1; CFI 0.953, TLI 0.774, RMSEA 0.068, SRMR 0.039). The model fit indices of the best-fit model indicated that the hypothesized model was well supported by the observed data (cut-offs commonly considered for a good model fit: CFI > 0.090, TLI > 0.090, RMSEA < 0.080, and SRMR < 0.060). In brief, we dropped paths that did not yield significant estimates. Based on modification indices (mi), we further added two correlation paths between smoking status and risk drinking (mi = 2444.854), and between non-occupational MVPA and LLDS (mi = 869.306). Additionally, several paths (e.g., TV watching to incident type 2 diabetes) were dropped because results from sensitivity analyses showed substantial changes in path coefficients, which suggested that these estimates were not robust. We present details of stepwise adjustments and reasons for changes in Supplementary Table 2.

Figure 2 presents the best-fit hypothesized model with standardized path coefficients. Paths related to age and sex are not shown in Fig. 2 but available in Supplementary Table 3. Among all modifiable risk factors included in the conceptual model (standardized *β*-coefficients are given in parentheses), waist circumference (0.214) had the strongest direct effect on type 2 diabetes, followed by HDL-cholesterol (− 0.134), triglycerides (0.096), income (− 0.074), blood pressure (0.055), diet quality (− 0.045), and smoking (0.035). Except for unhealthy sleep duration, education showed larger positive effects than income on all lifestyle behaviors. All included lifestyle behaviors were significantly associated with clinical markers, among which non-occupational MVPA, smoking, and TV watching yielded larger effect sizes. Risk drinking and smoking showed mixed effects on metabolic profiles. Almost all factors received strong direct effects from age and sex. In addition, correlations were found between BMI and waist circumference, between education and income, between triglycerides and HDL-cholesterol, between smoking status and risk drinking, and between diet quality and non-occupational MVPA.

For more information, please see Supplementary Table 3, which shows all standardized and unstandardized coefficients with standard errors for all paths.

Supplementary Table 4 shows the results of logistic regression model as a conventional approach for risk identification. The strongest effects were found for income group > 3000 euro/month (− 0.405), waist circumference (0.386), sex (women compared with men, 0.355), and HDL-cholesterol (− 0.339).

Results from sensitivity analyses showed consistent results, which indicated our estimates are robust. Compared with the main analysis, some variations were found when replacing incident type 2 diabetes by fasting glucose and HbA_1c_ measured at T4. Detailed discussions of sensitivity analyses are presented in Supplementary Text 3.

## Discussion

This study is the first that examined a broad range of key modifiable risk factors simultaneously in relation to incident type 2 diabetes using SEM. Our analysis quantified the complex pathways of these concomitant risk factors on the subsequent risk of developing type 2 diabetes, which provides valuable insights into the identification of priority prevention targets. Our results further extend knowledge of previous similar studies on prevalent prediabetes (Bardenheier et al., 2013) and prevalent type 2 diabetes (Roman-Urrestarazu et al., 2016) by incorporating four important lifestyle behavioral factors, i.e., smoking, TV watching, risk drinking, and unhealthy sleep duration.

### Interrelationships of Risk Factors

There are several key findings. First, of the two obesity indicators examined, large waist circumference was found to have a strong direct effect on type 2 diabetes. Our results highlight the importance of waist management, in addition to BMI control, for diabetes prevention in both clinical practice and public health interventions (Lee et al., 2018; Neeland et al., 2019). Second, blood lipids, assessed as a higher level of HDL-cholesterol and a lower level of triglycerides, had critical direct effects on lowering diabetes risk. Additionally, healthier lifestyle behaviors, especially watching less TV and engaging in more non-occupational MVPA, indirectly and favorably affected diabetes risk through the mediation of clinical markers (i.e., blood lipids and obesity status), indicating their equal importance in diabetes prevention.

For socioeconomic status, our analysis dissected the differential effects between education and income, showing that low education, rather than insufficient income, is the major upstream determinant of unhealthy lifestyle behaviors. In the context of The Netherlands, where the level of income inequality is relatively low, the effect of lower income on lifestyle behaviors may not predominantly be due to less access to healthy lifestyle resources. Instead, it is suggested that self-perceived control, attitudes, and social norms towards adopting a healthier lifestyle are more restrained among those with lower education (Stronks et al., 1997). Programs promoting healthy lifestyle should be complemented by additional elements to help people with lower education (Ball et al., 2012; Van der Lucht & Polder, 2010).

It is noteworthy that we observed direct effects of education on obesity status, as well as of income, diet quality, and smoking on type 2 diabetes. A cautious interpretation is warranted, as it cannot be excluded that the observed direct effects are in fact due to other, but unobserved, existing mediators or confounders, such as neighborhood deprivation (distal environmental factors) and chronic inflammation (proximal clinical biomarkers) (Dekker et al., 2020; Kivimäki et al., 2018; Zhu et al., 2021).

### Identification of Priority Prevention Targets

In terms of primary prevention, this simultaneous quantification of multiple risk factors and their intersecting pathways puts scattered evidence together and enables the identification of key upstream prevention targets for type 2 diabetes. Public health programs on these targets may have the potential to address as much of the broader risk profile as possible, particularly for those proximal clinical markers, for which pharmacological interventions may often be needed. Based on our results, (1) reducing large waist circumference may be prioritized as a main clinical target for diabetes prevention; (2) less TV watching time and more physical activity may be the main behavioral targets; and (3) better education may be the main societal target. Future studies are encouraged to examine the conceptual model in other populations.

It should be noted that the prevalence of type 2 diabetes at baseline in our population from the northern Netherlands (4.5%) is comparable to the average of upper-middle-income countries (5.6%), but lower than the average of high-income countries (7.9%) (Institute for Health Metrics and Evaluation, 2021). Regarding incidence, 1.6% of our study sample developed type 2 diabetes after a median follow-up of 41 months (230,259 person-years), which is translated into an incidence rate of 4.9 per 1000 person-years. In the literature, we found a wide range of incidence across different countries and cohorts, ranging from 2.6 per 1000 person-years in the UK Biobank study (Levy et al., 2021) to 11.4 per 1000 person-years in the American Multi-Ethnic Study of Atherosclerosis (Joseph et al., 2016). Despite the differences in cohort design and methodology that preclude direct comparisons, this high prevalence and incidence of type 2 diabetes worldwide call for us researchers to further work on curbing this global pandemic, especially by adopting innovative approaches to further build the evidence basis for the design of more effective public health programs (for detailed data, please see Supplementary Table 5).

### Strengths and Limitations

Conventional approaches for risk identification commonly estimate the total net effects of risk factors, but leave their interrelationships masked. We further illustrated this by comparing the results between using SEM and logistic regression model (Supplementary Table 4). More specifically, SEM clearly elucidated the extent to which education impacted on risk of type 2 diabetes through the mediation of lifestyle behaviors, while such information is unavailable in results from logistic regression models. Using SEM also avoids possible multiple testing of significance if each mediation pathway was modelled separately.

In our conceptual model, we did not develop latent variables as in previous similar studies (Bardenheier et al., 2013; Roman-Urrestarazu et al., 2016). Instead, we used single aggregate measures for diet and physical activity, and additionally added a correlation term between income and education. For diet and physical activity, our selected indicators are evidence-based and easy to apply to evaluation at population level (Byambasukh et al., 2020; Vinke et al., 2018). However, for latent variables, indicators were usually arbitrarily selected specifically to that study population, which may limit their generalizability. Nevertheless, we acknowledge that constructing a latent variable for lifestyle factors may help reduce measurement error. For effects of socioeconomic status, we clearly illustrated that the effects of income and education were different along the pathways to type 2 diabetes.

Our study also has some limitations. Even though we constructed the model in a prospective setting, the hypothesized pathways from socioeconomic status to clinical biomarkers are still of cross-sectional nature, although the lifestyle questionnaires were collected before the clinical measurements, and socioeconomic status was unlikely to change throughout the study period. An alternative conceptual model is also possible, even if model fit indices and sensitivity analyses indicate that our final model was well supported by the data observed. In addition, as the Lifelines cohort mainly consists of local Dutch participants, it may not be possible to extrapolate our results to other populations. Another limitation of this study is that misclassification could occur in the ascertainment of type 2 diabetes cases, since at T2 and T3 only self-reported data was available. We also regrettably do not have data on medication use during follow-ups to validate self-reported diagnosis of type 2 diabetes. However, as most cases were identified by objective laboratory measurements at T4, this limitation is unlikely to have introduced severe bias in our results. A final concern is that we regrettably could not analyze the potential impacts of lost to follow-up (23.2%) among eligible participants. Such attrition could affect our estimation, specifically for the pathways directly linked to type 2 diabetes status. Nonetheless, the baseline characteristics of those who had no follow-up data were comparable with the study population, except for some minor differences in education level (Supplementary Table 6). Simulation studies have shown that such attrition bias may only have limited influences on estimates of associations in regression analysis (Howe et al., 2013; Peters et al., 2012).

## Conclusions

This prospective study examined modifiable risk factors as a system in relation to incident type 2 diabetes through integrated pathways in a large population-based cohort. Quantifying the pathways of those modifiable risk factors using SEM may be a useful tool for the prioritization of prevention targets. Primary prevention strategies targeting proximal clinical risk factors should be complemented with public health initiatives that simultaneously address their corresponding upstream determinants. Regarding the current guideline for diabetes prevention, waist management in addition to BMI control (clinical level), as well as less TV watching in addition to more physical activity (behavioral level), may provide additional public health benefits. Better education would be the main societal goal for the prevention of type 2 diabetes.

## Supplementary Information

Below is the link to the electronic supplementary material.

Supplementary file1 (DOCX 207 KB)

## Data Availability

The manuscript is based on the data from the Lifelines cohort study. Lifelines adheres to standards for data availability. The data catalogue of the Lifelines cohort study is publicly accessible at www.lifelines.nl. All international researchers can obtain data at the Lifelines research office (research@lifelines.nl), for which a fee is required. The Lifelines research system allows access for reproducibility of the study results.

## Abbreviations

CFI: Comparative fit index
FFQ: Food frequency questionnaire
LLDS: Lifelines Diet Score
MVPA: Moderate-to-vigorous physical activity
RMSEA: Root mean square error of approximation
SEM: Structural equation modeling
SRMR: Standardized root mean square residual
TLI: Tucker-Lewis index

## References

[CR1] American Diabetes Association. (2020). 2. Classification and diagnosis of diabetes: Standards of medical care in diabetes—2020. *Diabetes Care*, *43*(Supplement 1), S14-S31. 10.2337/dc20-S002 10.2337/dc20-S002 31862745

[CR2] Astrup A (2001). Healthy lifestyles in Europe: Prevention of obesity and type II diabetes by diet and physical activity. Public Health Nutrition.

[CR3] Aune D, Norat T, Leitzmann M, Tonstad S, Vatten LJ (2015). Physical activity and the risk of type 2 diabetes: A systematic review and dose–response meta-analysis. European Journal of Epidemiology.

[CR4] Ball K, Abbott G, Cleland V, Timperio A, Thornton L, Mishra G, Crawford D (2012). Resilience to obesity among socioeconomically disadvantaged women: The READI study. International Journal of Obesity (london).

[CR5] Bardenheier BH, Bullard KM, Caspersen CJ, Cheng YJ, Gregg EW, Geiss LS (2013). A novel use of structural equation models to examine factors associated with prediabetes among adults aged 50 years and older: National Health and Nutrition Examination Survey 2001–2006. Diabetes Care.

[CR6] Black, A. E. (2000). Critical evaluation of energy intake using the Goldberg cut-off for energy intake:basal metabolic rate. A practical guide to its calculation, use and limitations. *International Journal of Obesity and Related Metabolic Disorders*, *24*(9), 1119–1130. 10.1038/sj.ijo.0801376 10.1038/sj.ijo.0801376 11033980

[CR7] Byambasukh O, Snieder H, Corpeleijn E (2020). Relation between leisure time, commuting, and occupational physical activity with blood pressure in 125 402 adults: The Lifelines Cohort. Journal of the American Heart Association.

[CR8] Cappuccio FP, D'Elia L, Strazzullo P, Miller MA (2010). Quantity and quality of sleep and incidence of type 2 diabetes: A systematic review and meta-analysis. Diabetes Care.

[CR9] Dekker LH, Rijnks RH, Navis GJ (2020). Regional variation in type 2 diabetes: Evidence from 137 820 adults on the role of neighbourhood body mass index. European Journal of Public Health.

[CR10] Després J-P, Lemieux I (2006). Abdominal obesity and metabolic syndrome. Nature.

[CR11] Duan MJ, Dekker LH, Carrero JJ, Navis G (2021). Blood lipids-related dietary patterns derived from reduced rank regression are associated with incident type 2 diabetes. Clinical Nutrition.

[CR12] Foster HM, Celis-Morales CA, Nicholl BI, Petermann-Rocha F, Pell JP, Gill JM, Mair FS (2018). The effect of socioeconomic deprivation on the association between an extended measurement of unhealthy lifestyle factors and health outcomes: A prospective analysis of the UK Biobank cohort. The Lancet Public Health.

[CR13] Howe LD, Tilling K, Galobardes B, Lawlor DA (2013). Loss to follow-up in cohort studies: Bias in estimates of socioeconomic inequalities. Epidemiology.

[CR14] Institute for Health Metrics and Evaluation. (2021). *Global Health Data Exchange (GHDx) query tool*. Global Burden of Diseases, Injuries, and Risk Factors Study. Retrieved 10 Nov 2021 from http://ghdx.healthdata.org/gbd-results-tool

[CR15] Jorgensen, T. D., Pornprasertmanit, S., Schoemann, A. M., Rosseel, Y., Miller, P., Quick, C., & Selig, J. (2019). Package ‘semTools’. https://cran.r-project.org/web/packages/semTools/semTools.pdf

[CR16] Joseph JJ, Echouffo-Tcheugui JB, Golden SH, Chen H, Jenny NS, Carnethon MR, Bertoni AG (2016). Physical activity, sedentary behaviors and the incidence of type 2 diabetes mellitus: The Multi-Ethnic Study of Atherosclerosis (MESA). BMJ Open Diabetes Research & Care.

[CR17] Kivimäki M, Vahtera J, Tabák AG, Halonen JI, Vineis P, Pentti J, Kähönen M (2018). Neighbourhood socioeconomic disadvantage, risk factors, and diabetes from childhood to middle age in the Young Finns Study: A cohort study. The Lancet Public Health.

[CR18] Klijs, B., Scholtens, S., Mandemakers, J. J., Snieder, H., Stolk, R. P., & Smidt, N. (2015). Representativeness of the LifeLines cohort study. *PLOS ONE*, *10*(9). 10.1371/journal.pone.0137203 10.1371/journal.pone.0137203 PMC4557968 26333164

[CR19] Kline, R. B. (2015). *Principles and practice of structural equation modeling* (4th ed.). Guilford publications.

[CR20] Knott, C., Bell, S., & Britton, A. (2015). Alcohol consumption and the risk of type 2 diabetes: A systematic review and dose-response meta-analysis of more than 1.9 million individuals from 38 observational studies. *Diabetes Care*, *38*(9), 1804–1812. 10.2337/dc15-0710 10.2337/dc15-0710 26294775

[CR21] Kruit JK, Brunham LR, Verchere CB, Hayden MR (2010). HDL and LDL cholesterol significantly influence β-cell function in type 2 diabetes mellitus. Current Opinion in Lipodology.

[CR22] Lakerveld J, Mackenbach J (2017). The upstream determinants of adult obesity. Obesity Facts.

[CR23] Lee DH, Keum N, Hu FB, Orav EJ, Rimm EB, Willett WC, Giovannucci EL (2018). Comparison of the association of predicted fat mass, body mass index, and other obesity indicators with type 2 diabetes risk: Two large prospective studies in US men and women. European Journal of Epidemiology.

[CR24] Levy RB, Rauber F, Chang K, Louzada M, Monteiro CA, Millett C, Vamos EP (2021). Ultra-processed food consumption and type 2 diabetes incidence: A prospective cohort study. Clinical Nutrition.

[CR25] Li, K.-H., Meng, X.-L., Raghunathan, T. E., & Rubin, D. B. (1991). Significance levels from repeated p-values with multiply-imputed data. *Statistica Sinica*, 65–92.

[CR26] Llavero-Valero, M., Escalada San Martín, J., Martínez-González, M. A., Alvarez-Mon, M. A., Alvarez-Alvarez, I., Martínez-González, J., & Bes-Rastrollo, M. (2021). Promoting exercise, reducing sedentarism or both for diabetes prevention: The “Seguimiento Universidad De Navarra” (SUN) cohort. *Nutrition, Metabolism & Cardiovascular Diseases*, *31*(2), 411-419.10.1016/j.numecd.2020.09.027 10.1016/j.numecd.2020.09.027 33234383

[CR27] Maghsoudi Z, Ghiasvand R, Salehi-Abargouei A (2016). Empirically derived dietary patterns and incident type 2 diabetes mellitus: A systematic review and meta-analysis on prospective observational studies. Public Health Nutrition.

[CR28] Maty SC, Everson-Rose SA, Haan MN, Raghunathan TE, Kaplan GA (2005). Education, income, occupation, and the 34-year incidence (1965–99) of type 2 diabetes in the Alameda County Study. International Journal of Epidemiology.

[CR29] Muthén, B., Du, S., Spisic, D., Muthén, B., & du Toit, S. (1997). Robust inference using weighted least squares and quadratic estimating equations in latent variable modeling with categorical and continuous outcomes. https://www.statmodel.com/wlscv.shtml

[CR30] Neeland IJ, Ross R, Després J-P, Matsuzawa Y, Yamashita S, Shai I, Arsenault B (2019). Visceral and ectopic fat, atherosclerosis, and cardiometabolic disease: A position statement. The Lancet Diabetes & Endocrinology.

[CR31] Pan A, Wang Y, Talaei M, Hu FB, Wu T (2015). Relation of active, passive, and quitting smoking with incident type 2 diabetes: A systematic review and meta-analysis. The Lancet Diabetes & Endocrinology.

[CR32] Patterson R, McNamara E, Tainio M, de Sá TH, Smith AD, Sharp SJ, Wijndaele K (2018). Sedentary behaviour and risk of all-cause, cardiovascular and cancer mortality, and incident type 2 diabetes: A systematic review and dose response meta-analysis. European Journal of Epidemiology.

[CR33] Peters SA, Bots ML, den Ruijter HM, Palmer MK, Grobbee DE, Crouse JR, Koffijberg H (2012). Multiple imputation of missing repeated outcome measurements did not add to linear mixed-effects models. Journal of Clinical Epidemiology.

[CR34] Roman-Urrestarazu A, Ali FMH, Reka H, Renwick MJ, Roman GD, Mossialos E (2016). Structural equation model for estimating risk factors in type 2 diabetes mellitus in a Middle Eastern setting: Evidence from the STEPS Qatar. BMJ Open Diabetes Research & Care.

[CR35] Rosseel, Y. (2012). Lavaan: An R package for structural equation modeling and more. Version 0.5–12 (BETA). *Journal of Statistical Software*, *48*(2), 1–36. 10.18637/jss.v048.i02

[CR36] Schofield W (1985). Predicting basal metabolic rate, new standards and review of previous work. Human Nutrition, Clinical Nutrition.

[CR37] Scholtens S, Smidt N, Swertz MA, Bakker SJ, Dotinga A, Vonk JM, Wolffenbuttel BH (2015). Cohort profile: LifeLines, a three-generation cohort study and biobank. International Journal of Epidemiology.

[CR38] Schulze MB, Hoffmann K, Manson JE, Willett WC, Meigs JB, Weikert C, Hu FB (2005). Dietary pattern, inflammation, and incidence of type 2 diabetes in women. American Journal of Clinical Nutrition.

[CR39] Streppel MT, de Vries JH, Meijboom S, Beekman M, de Craen AJ, Slagboom PE, Feskens EJ (2013). Relative validity of the food frequency questionnaire used to assess dietary intake in the Leiden Longevity Study. Nutrition Journal.

[CR40] Stronks K, van de Mheen HD, Looman CW, Mackenbach JP (1997). Cultural, material, and psychosocial correlates of the socioeconomic gradient in smoking behavior among adults. Preventive Medicine.

[CR41] UNESCO. (1997). *International Standard Classification of Education (ISCED) 1997*. Retrieved 01 Aug 2020 from http://www.unesco.org/education/information/nfsunesco/doc/isced_1997.htm

[CR42] Van Buuren S, Boshuizen HC, Knook DL (1999). Multiple imputation of missing blood pressure covariates in survival analysis. Statistics in Medicine.

[CR43] Van Buuren, S., & Groothuis-Oudshoorn, K. (2010). mice: Multivariate imputation by chained equations in R. *Journal of Statistical Software*, *45*(3), 1–67. 10.18637/jss.v045.i03

[CR44] Van der Lucht, F., & Polder, J. (2010). *Towards better health: The Dutch 2010 public health status and forecasts report*. https://www.rivm.nl/bibliotheek/rapporten/270061011.html

[CR45] Vinke PC, Corpeleijn E, Dekker LH, Jacobs DR, Navis G, Kromhout D (2018). Development of the food-based Lifelines Diet Score (LLDS) and its application in 129,369 Lifelines participants. European Journal of Clinical Nutrition.

[CR46] Vinke PC, Navis G, Kromhout D, Corpeleijn E (2020). Socio-economic disparities in the association of diet quality and type 2 diabetes incidence in the Dutch Lifelines cohort. EClinicalMedicine.

[CR47] von Eckardstein A, Widmann C (2014). High-density lipoprotein, beta cells, and diabetes. Cardiovascular Research.

[CR48] Wendel-Vos, G. W., Schuit, A. J., Saris, W. H., & Kromhout, D. (2003). Reproducibility and relative validity of the short questionnaire to assess health-enhancing physical activity. *Journal of Clinical Epidemiology*, *56*(12), 1163–1169. 10.1016/s0895-4356(03)00220-8 10.1016/s0895-4356(03)00220-8 14680666

[CR49] WHO Collaborating Centre for Drug Statistics Methodology, & Norwegian Institute of Public Health. (2020). *ATC/DDD index*. Retrieved 30 Aug 2020 from https://www.whocc.no/atc_ddd_index/

[CR50] Williams B, Mancia G, Spiering W, Agabiti Rosei E, Azizi M, Burnier M, Dominiczak A (2018). 2018 ESC/ESH Guidelines for the management of arterial hypertension: The Task Force for the Management of Arterial Hypertension of the European Society of Cardiology (ESC) and the European Society of Hypertension (ESH). Journal of Hypertension.

[CR51] Xia Y, Yang Y (2019). RMSEA, CFI, and TLI in structural equation modeling with ordered categorical data: The story they tell depends on the estimation methods. Behavior Research Methods.

[CR52] Zhu, Y., Duan, M. J., Riphagen, I. J., Minovic, I., Mierau, J. O., Carrero, J. J., & Dekker, L. H. (2021). Separate and combined effects of individual and neighbourhood socio-economic disadvantage on health-related lifestyle risk factors: A multilevel analysis. *International Journal of Epidemiology*, *50*(6), 1959-1969. 10.1093/ije/dyab079 10.1093/ije/dyab079 PMC8743118 34999857

