# Supplementary material for: Using Structural Equation Modeling to Untangle Pathways of Risk Factors Associated with Incident Type 2 Diabetes: the Lifelines Cohort Study
Source: Prev Sci. 2022 Mar 1;23(7):1090–100. doi: 10.1007/s11121-022-01357-5 (PMC9489566; doi:10.1007/s11121-022-01357-5)
Supplement: Supplementary file 1 — Supplementary file1 (DOCX 207 KB) [file 11121_2022_1357_MOESM1_ESM.docx]

**Using Structural Equation Modeling to Untangle Pathways of Risk Factors Associated with Incident Type 2 Diabetes: The Lifelines Cohort Study**

**Supplementary Files**

Ming-Jie Duan^1^; Louise H. Dekker^1,2^; Juan-Jesus Carrero^3^; Gerjan Navis^1^.

Corresponding author: Ming-Jie Duan, m.duan@umcg.nl

1 Department of Internal Medicine, University Medical Center Groningen, University of Groningen, Groningen, The Netherlands

2 National Institute for Public Health and the Environment, Bilthoven, The Netherlands

3 Department of Medical Epidemiology and Biostatistics, Karolinska Institutet, Stockholm, Sweden

**Supplementary Text 1** Information of recruitment strategy and the representativeness of the Lifelines study population

For the Lifelines cohort study, participants were included in the study between year 2006 and 2013. They were recruited through: (1) invitation from their registered general practitioners; (2) invitation from their family members who had already participated in the study; and (3) online self-registration on the Lifelines cohort study website; each of which accounted for 49%, 38%, and 13% of the total recruited study population, respectively. Approximately 25% of the invited individuals agreed to participate and were subsequently included in the study.

Previous studies have shown that the population sample of the Lifelines cohort is broadly representative of the general population in the north of The Netherlands. Compared with the local Dutch population registry data, participants from the Lifelines cohort study had a somewhat different demographic profile, e.g. more female, middle-aged, married, educated, and more Dutch native participants; however, there were rather minor differences observed for lifestyle behaviors, prevalence of various chronic diseases, and self-rated health. More detailed information about the Lifelines cohort study can be found elsewhere [1, 2].

[1] Klijs, B., Scholtens, S., Mandemakers, J. J., Snieder, H., Stolk, R. P., & Smidt, N. (2015). Representativeness of the LifeLines cohort study. PLOS ONE, 10(9), e0137203.

[2] Scholtens, S., Smidt, N., Swertz, M. A., *et al*. (2015). Cohort Profile: LifeLines, a three-generation cohort study and biobank. Int J Epidemiol, 44(4), 1172-1180.

**Supplementary Text 2** Methods for clinical measurements

For all clinical measurements, they were measured by trained research staff following standardized protocols. Blood samples were collected by venipuncture in a fasting state between 8 and 10 am. Serum level of glucose, HbA_1c_, HDL-cholesterol, and triglycerides were subsequently analyzed. HDL-cholesterol was determined by enzymatic colorimetric method; triglycerides was determined by colorimetric UV method; fasting glucose was determined by hexokinase method; and HbA_1c_ was determined by ion exchange chromatography. Except for HbA_1c_, all measurements were performed on Roche Modular P (Roche, Basel, Switzerland). Measurements of HbA_1c_ were performed on BioRad Variant 1 (BioRad, Hercules, USA).

Anthropometric measurements were performed without shoes, heavy clothing, and with empty pocket. Body weight was measured to 0.1kg by SECA 761 scale (Seca GmbH, Hamburg, Germany). Participant stepped on standard weighing scale and weight is recorded. Height was measured to 0.5cm using Frankfort Plane position by SECA 222 stadiometer (Seca GmbH, Hamburg, Germany). Participant was requested to stand with their back against the stadiometer, feet together, with the heels touching the wall, standing upright, and looking straight ahead. The horizontal headpiece is adjusted to rest on the head and height was recorded. In case the participant was not able to stand, arm span may be measured. Tip of one middle finger to the other was measured. Waist circumference was measured to 0.5cm by SECA 200/201 measuring tape (Seca GmbH, Hamburg, Germany). Participant was requested to stand upright, SECA 200/201 measurement tape is placed between lowest rib and the iliac crest around the bare stomach.

Systolic and diastolic blood pressure were measured 10 times within 10 minutes, and the average value of the last three readings were considered the blood pressure measured. Blood pressures were measured by Dynamap PRO 100V2 (GE Healthcare, Freiburg, Germany).

**Supplementary Text 3** Methods, results, and discussion of sensitivity analyses

Several sensitivity analyses were performed to test the robustness of our results. First, we replaced the outcome variable (incident type 2 diabetes) by fasting glucose and HbA_1c_ measured at T4. Second, we excluded participants lost to follow-up after T2 or T3, in an attempt to address the possible reverse causation caused by short follow-up time. Third, education and income were replaced by a latent variable of socioeconomic status measured by education and income [1]. Fourth, the SEM analysis was repeated 3 times on a random half sample of the study population. Fifth, the model was examined with maximum likelihood estimator, and the fit indices were compared with the WLSMV estimator to check possible model misspecification [2]. Finally, two other methods for handling missing data were performed, i.e. applying the full information maximum likelihood estimator and complete case analysis with WLSMV estimator [3,4].

Results from sensitivity analyses were compared with our best-fit model to assess the robustness of our main SEM analysis. In general, these results (data not shown) are consistent with our main analysis. First, results were basically unchanged when we excluded participants lost to follow-up after T2 or T3. Second, replacing income and education by a latent construct of socio-economic status yielded similar results as well, except for path coefficients from socio-economic status to lifestyle behaviors. Third, we obtained similar path coefficients when analyzing the random half sample. Fourth, findings were also similar when the complete case analysis was performed, although standard errors for path coefficients were somewhat larger. Fifth, using full information maximum likelihood estimator yielded similar results as well, except for path coefficients to incident type 2 diabetes, which was due to the fact that this estimator cannot efficiently handle binary endogenous variable. Sixth, the SEM analysis of the two alternative hypothesized models using fasting glucose or HbA_1c_ as outcomes provided consistent estimates, although some variations were found on HDL-cholesterol and income (please see table and discussion below). For all sensitivity analyses (including using other estimators), model fit indices did not show substantial differences.

When analyzing the two alternative models using fasting glucose or HbA_1c_ as outcome, we found that the effect sizes of HDL-cholesterol and income showed some variations compared with our main results, while there are also some other minor variations observed, such as the direct effect of blood pressure on HbA_1c_. We consider these variations acceptable and do not affect out conclusions. There are several reasons that may explain these variations. In brief, one major reason we consider possible is the treatment effect for those who had been diagnosed with type 2 diabetes, as their HbA_1c_ and fasting glucose level may be influenced by their long-term daily medication intake and lifestyle intervention, particularly HbA_1c_ - serving as a long-term indicator for glycemic control in people with diabetes. It is also conceivable that the diagnostic criteria for type 2 diabetes is a composite of self-reported diabetes, fasting glucose measurement, and HbA_1c_ measurement, with which the associations may vary according to different risk factors, reflecting different pathways to the development of the disease. Also, we could not exclude the possibility of other uncaptured variables that may explain these variations.

[1] Hagger-Johnson, Gareth, *et al*. Childhood socioeconomic status and adult health: comparing formative and reflective models in the Aberdeen Children of the 1950s Study (prospective cohort study). J Epidemiol Community Health 65.11 (2011): 1024-1029.

[2] Xia, Yan, and Yanyun Yang. RMSEA, CFI, and TLI in structural equation modeling with ordered categorical data: The story they tell depends on the estimation methods. Behav Res Methods 51.1 (2019): 409-428.

[3] Kline RB. Principles and practice of structural equation modeling. 4th ed. New York, NY, USA (2015): Guilford publications.

[4] Enders, Craig K., and Deborah L. Bandalos. The relative performance of full information maximum likelihood estimation for missing data in structural equation models. Structural equation modeling 8.3 (2001): 430-457.

**Supplementary Text 3 (Table)** Comparisons of estimates of two alternative hypothesized models with fasting glucose and HbA_1c_ measured at the second assessment (T4) as outcome (*n* = 68,649)

| **Outcome variable** | **Regressors** | **Unstandardized path coefficients (standard error)** | **Standardized path coefficients** |
| --- | --- | --- | --- |
| **Type 2 diabetes status** | Age | 0.017 (0.001) | 0.153 |
|  | Sex | 0.220 (0.027) | 0.105 |
|  | Smoking status | 0.035 (0.014) | 0.035 |
|  | LLDS | -0.046 (0.012) | -0.045 |
|  | Income | -0.075 (0.014) | -0.074 |
|  | Triglycerides | 0.099 (0.014) | 0.096 |
|  | HDL-cholesterol | -0.138 (0.014) | -0.134 |
|  | Waist circumference | 0.221 (0.013) | 0.214 |
|  | Blood pressure | 0.056 (0.013) | 0.055 |
| **Fasting glucose (T4)** | Age | 0.010 (0.000) | 0.097 |
|  | Sex | -0.110 (0.009) | -0.055 |
|  | Smoking status | 0.031 (0.004) | 0.031 |
|  | LLDS | -0.027 (0.004) | -0.027 |
|  | Income | -0.019 (0.005) | -0.019 |
|  | Triglycerides | 0.096 (0.002) | 0.096 |
|  | HDL-cholesterol | -0.058 (0.003) | -0.058 |
|  | Waist circumference | 0.237 (0.002) | 0.234 |
|  | Blood pressure | 0.064 (0.003) | 0.064 |
| **HbA_1c_ (T4)** | Age | 0.032 (0.000) | 0.304 |
|  | Sex | 0.201 (0.010) | 0.100 |
|  | Smoking status | 0.061 (0.004) | 0.063 |
|  | LLDS | -0.032 (0.004) | -0.032 |
|  | Income | -0.046 (0.005) | -0.047 |
|  | Triglycerides | 0.077 (0.004) | 0.077 |
|  | HDL-cholesterol | -0.030 (0.005) | -0.031 |
|  | Waist circumference | 0.126 (0.005) | 0.125 |
|  | Blood pressure | 0.016 (0.004) | 0.017 |

**Supplementary Fig. 1** Timeline of data collection of the Lifelines cohort study


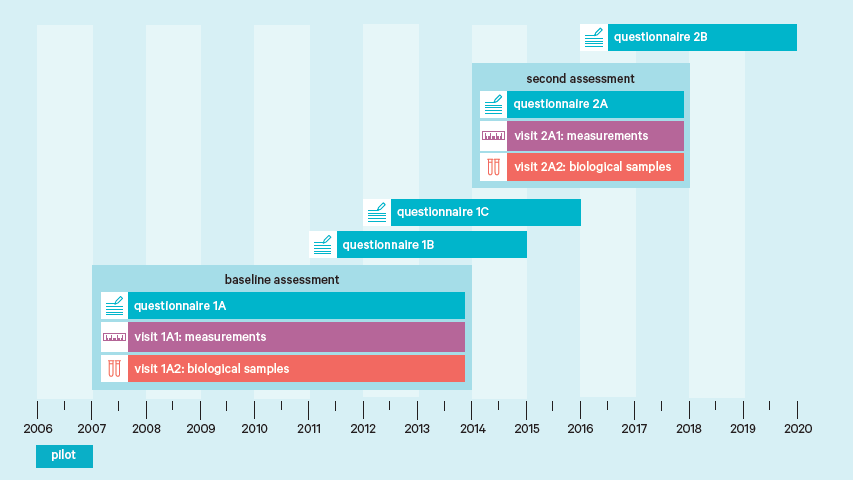


Corresponding to the time point mentioned in the manuscript, T1-baseline assessment, T2-questionnaire 1B, T3-questionnaire 1C, and T4-second assessment.

Detailed information can be found at: https://www.lifelines.nl/researcher/about-lifelines

**Supplementary Fig. 2** Study flow chart with inclusion and exclusion criteria


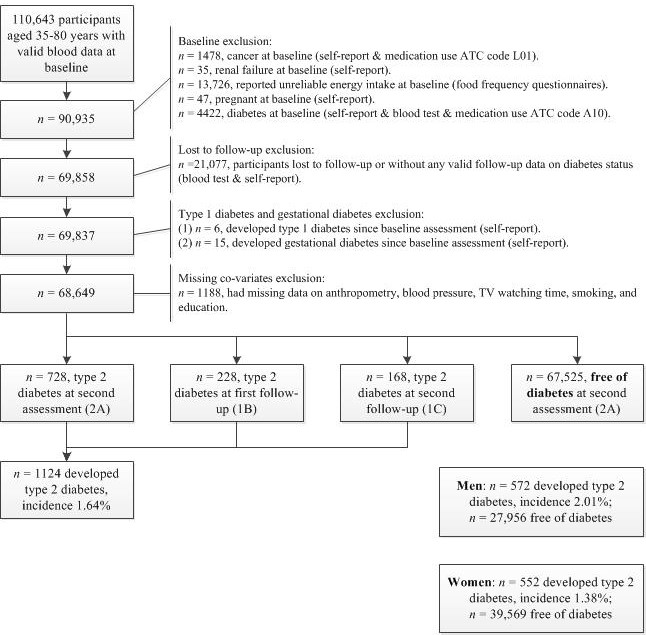


**Supplementary Table 1** Summary of supporting literature for pathways investigated in the conceptual model for incident type 2 diabetes

| **Pathways investigated in the conceptual model** | **DOI** |
| --- | --- |
| **Previous Structural equation modeling studies** | |
| Bardenheier, Barbara H., et al. A novel use of structural equation models to examine factors associated with prediabetes among adults aged 50 years and older: National Health and Nutrition Examination Survey 2001–2006. Diabetes Care 36.9 (2013): 2655-2662. | 10.2337/dc12-2608 |
| Roman-Urrestarazu, Andres, et al. Structural equation model for estimating risk factors in type 2 diabetes mellitus in a Middle Eastern setting: evidence from the STEPS Qatar. BMJ Open Diabetes Research and Care 4.1 (2016). | 10.1136/bmjdrc-2016-000231 |
| Paudel, Susan, et al. The contribution of physical inactivity and socioeconomic factors to type 2 diabetes in Nepal: a structural equation modelling analysis. Nutrition, Metabolism and Cardiovascular Diseases (2020). | 10.1016/j.numecd.2020.06.003 |
| **Socio-economic status ~ Lifestyle** | |
| Foster, Hamish ME, et al. The effect of socioeconomic deprivation on the association between an extended measurement of unhealthy lifestyle factors and health outcomes: a prospective analysis of the UK Biobank cohort. The Lancet Public Health 3.12 (2018): e576-e585. | 10.1016/S2468-2667(18)30200-7 |
| Beenackers, Marielle A., et al. Socioeconomic inequalities in occupational, leisure-time, and transport related physical activity among European adults: a systematic review. International Journal of Behavioral Nutrition and Physical Activity 9.1 (2012): 1-23. | 10.1186/1479-5868-9-116 |
| Andrade-Gómez, Elena, et al. Watching TV has a distinct sociodemographic and lifestyle profile compared with other sedentary behaviors: A nationwide population-based study. PLOS ONE 12.12 (2017): e0188836. | 10.1371/journal.pone.0188836 |
| Stronks, Karien, et al. Cultural, material, and psychosocial correlates of the socioeconomic gradient in smoking behavior among adults. Preventive Medicine 26.5 (1997): 754-766. | 10.1006/pmed.1997.0174 |
| Darmon, Nicole, and Adam Drewnowski. Does social class predict diet quality?. The American Journal of Clinical Nutrition 87.5 (2008): 1107-1117. | 10.1093/ajcn/87.5.1107 |
| Thiele, Silke, et al. "Determinants of diet quality." Public Health Nutrition 7.1 (2004): 29-37. | 10.1079/PHN2003516 |
| Hulshof, K. F. A. M., et al. "Socio-economic status, dietary intake and 10 y trends: the Dutch National Food Consumption Survey." European Journal of Clinical Nutrition 57.1 (2003): 128-137. | 10.1038/sj.ejcn.1601503 |
| **Socio-economic status ~ Obesity status** | |
| León-Muñoz, Luz Maria, et al. Contribution of lifestyle factors to educational differences in abdominal obesity among the adult population. Clinical Nutrition 33.5 (2014): 836-843. | 10.1016/j.clnu.2013.10.013 |
| Ball, K., et al. Resilience to obesity among socioeconomically disadvantaged women: the READI study. International Journal of Obesity 36.6 (2012): 855-865. | 10.1038/ijo.2011.183 |
| **Socio-economic status ~ Blood pressure** | |
| Leng, Bing, et al. Socioeconomic status and hypertension: a meta-analysis. Journal of Hypertension 33.2 (2015): 221-229. | 10.1097/HJH.0000000000000428 |
| **Socio-economic status ~ Type 2 diabetes** | |
| Stringhini, Silvia, et al. Contribution of modifiable risk factors to social inequalities in type 2 diabetes: prospective Whitehall II cohort study. BMJ 345 (2012): e5452. | 10.1136/bmj.e5452 |
| Vinke, Petra C., et al. Socio-economic disparities in the association of diet quality and type 2 diabetes incidence in the dutch lifelines cohort. EClinicalMedicine 19 (2020): 100252. | 10.1016/j.eclinm.2019.100252 |
| Williams, Emily D., et al. Health behaviours, socioeconomic status and diabetes incidence: the Australian Diabetes Obesity and Lifestyle Study (AusDiab). Diabetologia 53.12 (2010): 2538-2545. | 10.1007/s00125-010-1888-4 |
| Lee, Timothy C., et al. Socioeconomic status and incident type 2 diabetes mellitus: data from the Women's Health Study. PLOS ONE 6.12 (2011): e27670. | 10.1371/journal.pone.0027670 |
| **Physical activity and TV watching ~ Blood lipids, Obesity status, and Blood pressure** | |
| Healy, Genevieve N., et al. Objectively measured sedentary time, physical activity, and metabolic risk: the Australian Diabetes, Obesity and Lifestyle Study (AusDiab). Diabetes Care 31.2 (2008): 369-371. | 10.2337/dc07-1795 |
| Smith, Lee, Abigail Fisher, and Mark Hamer. Television viewing time and risk of incident obesity and central obesity: the English longitudinal study of ageing. BMC Obesity 2.1 (2015): 1-5. | 10.1186/s40608-015-0042-8 |
| Crichton, Georgina E., and Ala’A. Alkerwi. Physical activity, sedentary behavior time and lipid levels in the Observation of Cardiovascular Risk Factors in Luxembourg study. Lipids in Health and Disease 14.1 (2015): 1-9. | 10.1186/s12944-015-0085-3 |
| Kronenberg, Florian, et al. Influence of leisure time physical activity and television watching on atherosclerosis risk factors in the NHLBI Family Heart Study. Atherosclerosis 153.2 (2000): 433-443. | 10.1016/S0021-9150(00)00426-3 |
| Stamatakis, Emmanuel, et al. Moderate-to-vigorous physical activity and sedentary behaviours in relation to body mass index-defined and waist circumference-defined obesity. British Journal of Nutrition 101.5 (2008): 765-773. | 10.1017/S0007114508035939 |
| **Diet ~ Blood lipids, Obesity status, and Blood pressure** | |
| Duan, Ming-Jie, et al. Blood lipids-related dietary patterns derived from reduced rank regression are associated with incident type 2 diabetes. Clinical Nutrition 40.7 (2021): 4712-4719. | 10.1016/j.clnu.2021.04.046 |
| Park, Yong-Moon, et al. Obesity mediates the association between Mediterranean diet consumption and insulin resistance and inflammation in US adults. The Journal of Nutrition 147.4 (2017): 563-571. | 10.3945/jn.116.243543 |
| McNaughton, Sarah A., et al. Food patterns associated with blood lipids are predictive of coronary heart disease: the Whitehall II study. British Journal of Nutrition 102.4 (2009): 619-624. | 10.1017/S0007114509243030 |
| Heidemann, C., et al. A dietary pattern protective against type 2 diabetes in the European Prospective Investigation into Cancer and Nutrition (EPIC)—Potsdam Study cohort. Diabetologia 48.6 (2005): 1126-1134. | 10.1007/s00125-005-1743-1 |
| Sonestedt, Emily, et al. Diet quality and change in blood lipids during 16 years of follow-up and their interaction with genetic risk for dyslipidemia. Nutrients 8.5 (2016): 274. | 10.3390/nu8050274 |
| Buijsse, Brian, et al. Plasma ascorbic acid, a priori diet quality score, and incident hypertension: a prospective cohort study. PLOS ONE 10.12 (2015): e0144920. | 10.1371/journal.pone.0144920 |
| **Smoking ~ Blood lipids, Obesity status, and Blood pressure** | |
| Campbell, Sara Chelland, et al. Smoking and smoking cessation—the relationship between cardiovascular disease and lipoprotein metabolism: a review. Atherosclerosis 201.2 (2008): 225-235. | 10.1016/j.atherosclerosis.2008.04.046 |
| Chiolero, Arnaud, et al. Consequences of smoking for body weight, body fat distribution, and insulin resistance. The American Journal of Clinical Nutrition 87.4 (2008): 801-809. | 10.1093/ajcn/87.4.801 |
| Li, Guoju, et al. The association between smoking and blood pressure in men: a cross-sectional study. BMC Public Health 17.1 (2017): 797. | 10.1186/s12889-017-4802-x |
| Primatesta, Paola, et al. Association between smoking and blood pressure: evidence from the health survey for England. Hypertension 37.2 (2001): 187-193. | 10.1161/01.HYP.37.2.187 |
| **Unhealthy sleep duration ~ Blood lipids, Obesity status, and Blood pressure** | |
| Smiley, Abbas, et al. The association between sleep duration and lipid profiles: the NHANES 2013–2014. Journal of Diabetes & Metabolic Disorders 18.2 (2019): 315-322. | 10.1007/s40200-019-00415-0 |
| Bayon, Virginie, et al. Sleep debt and obesity. Annals of Medicine 46.5 (2014): 264-272. | 10.3109/07853890.2014.931103 |
| Cappuccio, Francesco P., and Michelle A. Miller. Sleep and cardio-metabolic disease. Current Cardiology Reports 19.11 (2017): 1-9. | 10.1007/s11886-017-0916-0 |
| **Risk drinking ~ Blood lipids, Obesity status, and Blood pressure** | |
| Fernández-Solà, Joaquim. Cardiovascular risks and benefits of moderate and heavy alcohol consumption. Nature Reviews Cardiology 12.10 (2015): 576-587. | 10.1038/nrcardio.2015.91 |
| Traversy, Gregory, and Jean-Philippe Chaput. Alcohol consumption and obesity: an update. Current Obesity Reports 4.1 (2015): 122-130. | 10.1007/s13679-014-0129-4 |
| **Lifestyle ~ Type 2 diabetes** | |
| Zhang, Yanbo, et al. Combined lifestyle factors and risk of incident type 2 diabetes and prognosis among individuals with type 2 diabetes: a systematic review and meta-analysis of prospective cohort studies. Diabetologia 63.1 (2020): 21-33. | 10.1007/s00125-019-04985-9 |
| Hu, Frank B., et al. Diet, lifestyle, and the risk of type 2 diabetes mellitus in women. New England Journal of Medicine 345.11 (2001): 790-797. | 10.1056/NEJMoa010492 |
| Maghsoudi, Zahra, et al. Empirically derived dietary patterns and incident type 2 diabetes mellitus: a systematic review and meta-analysis on prospective observational studies. Public Health Nutrition 19.2 (2016): 230-241. | 10.1017/S1368980015001251 |
| Pan, An, et al. Relation of active, passive, and quitting smoking with incident type 2 diabetes: a systematic review and meta-analysis. The Lancet Diabetes & Endocrinology 3.12 (2015): 958-967. | 10.1016/S2213-8587(15)00316-2 |
| Aune, Dagfinn, et al. Physical activity and the risk of type 2 diabetes: a systematic review and dose–response meta-analysis. European Journal of Epidemiology (2015): 529-542. | 10.1007/s10654-015-0056-z |
| Llavero-Valero, María, et al. Promoting exercise, reducing sedentarism or both for diabetes prevention: The “Seguimiento Universidad De Navarra”(SUN) cohort. Nutrition, Metabolism and Cardiovascular Diseases (2020). | 10.1016/j.numecd.2020.09.027 |
| Cappuccio, Francesco P., et al. Quantity and quality of sleep and incidence of type 2 diabetes: a systematic review and meta-analysis. Diabetes Care 33.2 (2010): 414-420. | 10.2337/dc09-1124 |
| Shan, Zhilei, et al. Sleep duration and risk of type 2 diabetes: a meta-analysis of prospective studies. Diabetes Care 38.3 (2015): 529-537. | 10.2337/dc14-2073 |
| Knott, Craig, Steven Bell, and Annie Britton. Alcohol consumption and the risk of type 2 diabetes: a systematic review and dose-response meta-analysis of more than 1.9 million individuals from 38 observational studies. Diabetes Care 38.9 (2015): 1804-1812. | 10.2337/dc15-0710 |
| Patterson, Richard, et al. Sedentary behaviour and risk of all-cause, cardiovascular and cancer mortality, and incident type 2 diabetes: a systematic review and dose response meta-analysis. European Journal of Epidemiology (2018): 811-829. | 10.1007/s10654-018-0380-1 |
| Astrup, Arne. Healthy lifestyles in Europe: prevention of obesity and type II diabetes by diet and physical activity. Public Health Nutrition 4.2b (2001): 499-515. | 10.1079/PHN2001136 |
| Wannamethee, S. Goya, et al. Physical activity, metabolic factors, and the incidence of coronary heart disease and type 2 diabetes. Archives of Internal Medicine 160.14 (2000): 2108-2116. | 10.1001/archinte.160.14.2108 |
| **Blood lipids ~ Obesity status** | |
| Klop, Boudewijn, et al. Dyslipidemia in obesity: mechanisms and potential targets. Nutrients 5.4 (2013): 1218-1240. | 10.3390/nu5041218 |
| Després, Jean-Pierre, and Isabelle Lemieux. Abdominal obesity and metabolic syndrome. Nature 444.7121 (2006): 881-887. | 10.1038/nature05488 |
| **Blood lipids ~ Blood pressure** | |
| Strazzullo, Pasquale, et al. Do statins reduce blood pressure? A meta-analysis of randomized, controlled trials. Hypertension 49.4 (2007): 792-798. | 10.1161/01.HYP.0000259737.43916.42 |
| Mente, Andrew, et al. Association of dietary nutrients with blood lipids and blood pressure in 18 countries: a cross-sectional analysis from the PURE study. The Lancet Diabetes & Endocrinology 5.10 (2017): 774-787. | 10.1016/S2213-8587(17)30283-8 |
| Geleijnse, Johanna M., et al. Blood pressure response to fish oil supplementation: metaregression analysis of randomized trials. Journal of Hypertension (2002): 1493-1499. | 10.1097/00004872-200208000-00010 |
| **Blood lipids ~ Type 2 diabetes** |  |
| von Eckardstein, Arnold, and Christian Widmann. High-density lipoprotein, beta cells, and diabetes. Cardiovascular Research 103.3 (2014): 384-394. | 10.1093/cvr/cvu143 |
| Kruit, Janine K., et al. HDL and LDL cholesterol significantly influence β-cell function in type 2 diabetes mellitus. Current Opinion in Lipidology 21.3 (2010): 178-185. | 10.1097/MOL.0b013e328339387b |
| Rutti, Sabine, et al. Low-and high-density lipoproteins modulate function, apoptosis, and proliferation of primary human and murine pancreatic β-cells. Endocrinology 150.10 (2009): 4521-4530. | 10.1210/en.2009-0252 |
| **Obesity status ~ Blood pressure and Type 2 diabetes** | |
| Neeland, Ian J., et al. Visceral and ectopic fat, atherosclerosis, and cardiometabolic disease: a position statement. The Lancet Diabetes & Endocrinology 7.9 (2019): 715-725. | 10.1016/S2213-8587(19)30084-1 |
| Lee, Dong Hoon, et al. Comparison of the association of predicted fat mass, body mass index, and other obesity indicators with type 2 diabetes risk: two large prospective studies in US men and women. European Journal of Epidemiology 33.11 (2018): 1113-1123. | 10.1007/s10654-018-0433-5 |
| Mertens, Ilse L., and Luc F. Van Gaal. Overweight, obesity, and blood pressure: the effects of modest weight reduction. Obesity research 8.3 (2000): 270-278. | 10.1038/oby.2000.32 |
| Tanaka, Masami. Improving obesity and blood pressure. Hypertension Research 43.2 (2020): 79-89. | 10.1038/s41440-019-0348-x |
| **Blood pressure ~ Type 2 diabetes** | |
| Cheung, Bernard MY. The hypertension–diabetes continuum. Journal of Cardiovascular Pharmacology 55.4 (2010): 333-339. | 10.1097/FJC.0b013e3181d26430 |
| Smulyan, Harold, et al. Hypertension, diabetes type II, and their association: role of arterial stiffness. American Journal of Hypertension 29.1 (2016): 5-13. | 10.1093/ajh/hpv107 |
| **Other relevant studies** | |
| Zheng, Yan, et al. Global aetiology and epidemiology of type 2 diabetes mellitus and its complications. Nature Reviews Endocrinology 14.2 (2018): 88. | 10.1038/nrendo.2017.151 |
| Abbasi, Ali, et al. Prediction models for risk of developing type 2 diabetes: systematic literature search and independent external validation study. British Medical Journal 345 (2012). | 10.1136/bmj.e5900 |
| Whelton, Seamus P., et al. Effect of dietary fiber intake on blood pressure: a meta-analysis of randomized, controlled clinical trials. Journal of Hypertension (2005): 475-481. | 10.1097/01.hjh.0000160199.51158.cf |

**Supplementary Table 2** Stepwise adjustments for the hypothesized conceptual model using SEM (*n* = 68,649)

| **Model versions^a^** | **Dropped pathways^b^** | **Reasons** |
| --- | --- | --- |
| **Original model**  (*df*=19, CFI=0.953, TLI=0.774, RMSEA=0.068, SRMR=0.039) | Sex to unhealthy sleep duration | Insignificant estimates, *p* > 0.05 |
|  | LLDS to BMI |  |
|  | Risk drinking to BMI |  |
|  | Sleep to blood pressure |  |
|  | Income to blood pressure |  |
|  | BMI to type 2 diabetes |  |
|  | Risk drinking to type 2 diabetes |  |
|  | Sleep to type 2 diabetes |  |
|  | Education to type 2 diabetes |  |
| **Second model**  (*df*=28, CFI=0.951, TLI=0.841, RMSEA=0.057, SRMR=0.040) | **Correlation added:**  Smoking and risk drinking | Modification index (mi = 2444.854) |
| **Third model**  (*df*=27, CFI=0.973, TLI=0.909, RMSEA=0.043, SRMR=0.026) | Income to LLDS | Insignificant estimate, *p* > 0.05 |
| **Fourth model**  (*df*=28, CFI=0.973, TLI=0.912, RMSEA=0.042, SRMR=0.026) | **Correlation added:**  LLDS and MVPA | Modification index (mi = 877.421) |
| **Fifth model**  (*df*=27, CFI=0.981, TLI=0.937, RMSEA=0.036, SRMR=0.023) | Income to MVPA | Insignificant estimates, *p* > 0.05 |
|  | MVPA to type 2 diabetes |  |
|  |  |  |
| **Sixth model**  (*df*=29, CFI=0.981, TLI=0.941, RMSEA=0.035, SRMR=0.023) | Sex to TV watching time | Sensitivity analyses^c^ |
|  | Income to BMI |  |
|  | TV watching time to type 2 diabetes |  |
|  | Sleep to BMI |  |
|  | Sleep to waist circumference |  |
| **Final best-fit model**  (*df*=34, CFI=0.981, TLI=0.949, RMSEA=0.032, SRMR=0.023) |  |  |

^a^ CFI denotes comparative fit index; TLI denotes Tucker-Lewis index; RMSEA denotes root mean square error of approximation; SRMR denotes standardized root mean square residual; and *df* denotes degree of freedom of the tested model.

^b^ LLDS denotes Lifelines diet score; MVPA denotes non-occupational moderate-to-vigorous physical activity; and sleep denotes unhealthy sleep duration.

^c^ Results from sensitivity analyses consistently showed that a particular path was not significant or showed substantial changes in path coefficients, which suggested that this path was not robust.

**Detailed description of the procedures for the stepwise adjustments:**

In brief, we followed the following procedures (as a cycle) to reach the final best-fitting model. First, we dropped the paths that did not yield significant path estimate (*p* < 0.05 was considered significant). Second, we checked model modification indices (mi) to see if we added a certain path, to what degree the model fit would change (based on χ^2^ statistics). Third, we cross-checked with sensitivity analyses to make sure all the paths remained and their estimates were robust. During these procedures, we always cross-checked with literature to make sure these adjustments make sense. We repeated this round until we reached the final best-fitting model as presented.

In our analysis, we followed the abovementioned procedures and we did observe after each round of adjustments, the model fit indices improved. Based on model modification indices (mi), we added two paths - the correlation term between smoking and risk drinking, and the correlation term between Lifeline diet score (LLDS) and non-occupational moderate to vigorous physical activity level (MVPA). These two correlation paths were added because: (1) the modification indices were considered large enough to make sense; (2) after adding such paths, the standardized estimated path coefficients were larger than 0.100, the value of which was generally considered make sense; and (3) there are supporting evidence showing that lifestyle factors tended to cluster [1]. We also checked the Spearman correlation coefficients among these variables and the values supported this model change: between risk drinking and smoking *r* = 0.1951, *p* < 0.001; between LLDS and non-occupational MVPA *r* = 0.1358, *p* < 0.001.

In general, these adjustments were made considering the following reasons: (1) to achieve a parsimonious model with larger degree of freedom; (2) to avoid statistically significant results by chance because of large sample size; and (3) to be consistent with previous evidence and to ensure the scientific plausibility of the hypothesized conceptual model.

[1] Hendryx, M., Dinh, P., Chow, A., *et al*. Lifestyle and Psychosocial Patterns and Diabetes Incidence Among Women with and Without Obesity: a Prospective Latent Class Analysis. Prevention Science: the Official Journal of the Society for Prevention Research, 2020: 21:850-860.

**Supplementary Table 3** Estimates of the best-fit model^a^

|  | | **Unstandardized estimates**  **(standard error)** | **Standardized estimates** |
| --- | --- | --- | --- |
| **Direct effects** | **Regressors** | **Path coefficients** | **Standardized path coefficients** |
| **Income** | Age | -0.015 (0.000) | -0.141 |
|  | Sex | -0.247 (0.009) | -0.120 |
| **Education** | Age | -0.024 (0.000) | -0.223 |
|  | Sex | -0.139 (0.009) | -0.066 |
| **Smoking status** | Age | 0.003 (0.001) | 0.024 |
|  | Sex | -0.123 (0.009) | -0.059 |
|  | Education | -0.192 (0.006) | -0.193 |
|  | Income | -0.022 (0.005) | -0.022 |
| **LLDS** | Age | 0.030 (0.000) | 0.285 |
|  | Sex | 0.053 (0.008) | 0.026 |
|  | Education | 0.229 (0.004) | 0.235 |
| **TV watching** | Age | 0.011 (0.000) | 0.105 |
|  | Education | -0.283 (0.004) | -0.290 |
|  | Income | -0.069 (0.004) | -0.070 |
| **MVPA** | Age | 0.016 (0.000) | 0.151 |
|  | Sex | 0.103 (0.008) | 0.050 |
|  | Education | 0.121 (0.005) | 0.122 |
| **Risk drinking** | Age | 0.013 (0.001) | 0.117 |
|  | Sex | -0.593 (0.012) | -0.277 |
|  | Education | -0.037 (0.007) | -0.036 |
|  | Income | 0.089 (0.007) | 0.085 |
| **Unhealthy sleep duration** | Age | 0.008 (0.001) | 0.073 |
|  | Education | -0.094 (0.012) | -0.095 |
|  | Income | -0.138 (0.011) | -0.137 |
| **Triglycerides** | Age | 0.009 (0.000) | 0.090 |
|  | Sex | -0.542 (0.008) | -0.267 |
|  | Smoking status | 0.099 (0.004) | 0.101 |
|  | LLDS | -0.061 (0.004) | -0.061 |
|  | MVPA | -0.088 (0.004) | -0.089 |
|  | TV watching | 0.106 (0.004) | 0.106 |
|  | Risk drinking | 0.032 (0.006) | 0.033 |
|  | Unhealthy sleep duration | 0.075 (0.008) | 0.077 |
| **HDL-cholesterol** | Age | 0.011 (0.000) | 0.103 |
|  | Sex | 0.969 (0.008) | 0.477 |
|  | Smoking status | -0.125 (0.004) | -0.128 |
|  | LLDS | 0.049 (0.004) | 0.049 |
|  | MVPA | 0.104 (0.004) | 0.106 |
|  | TV watching | -0.081 (0.003) | -0.081 |
|  | Risk drinking | 0.191 (0.006) | 0.201 |
|  | Unhealthy sleep duration | -0.054 (0.008) | -0.055 |
| **Waist circumference** | Age | 0.012 (0.000) | 0.113 |
|  | Sex | -0.410 (0.008) | -0.202 |
|  | Smoking status | -0.036 (0.004) | -0.037 |
|  | LLDS | -0.020 (0.003) | -0.020 |
|  | MVPA | -0.062 (0.003) | -0.063 |
|  | TV watching | 0.084 (0.003) | 0.084 |
|  | Risk drinking | 0.060 (0.006) | 0.063 |
|  | Education | -0.067 (0.005) | -0.069 |
|  | Income | -0.024 (0.004) | -0.024 |
|  | Triglycerides | 0.165 (0.004) | 0.165 |
|  | HDL-cholesterol | -0.271 (0.004) | -0.271 |
| **BMI** | Age | 0.004 (0.000) | 0.038 |
|  | Sex | 0.148 (0.008) | 0.073 |
|  | Smoking status | -0.052 (0.004) | -0.054 |
|  | MVPA | -0.038 (0.004) | -0.039 |
|  | TV watching | 0.099 (0.004) | 0.098 |
|  | Education | -0.093 (0.005) | -0.095 |
|  | Triglycerides | 0.176 (0.004) | 0.176 |
|  | HDL-cholesterol | -0.260 (0.004) | -0.260 |
| **Blood pressure** | Age | 0.025 (0.000) | 0.235 |
|  | Sex | -0.327 (0.009) | -0.161 |
|  | Smoking status | -0.057 (0.005) | -0.058 |
|  | LLDS | -0.019 (0.004) | -0.019 |
|  | MVPA | -0.018 (0.004) | -0.018 |
|  | TV watching | 0.024 (0.004) | 0.024 |
|  | Risk drinking | 0.062 (0.006) | 0.066 |
|  | Education | -0.054 (0.005) | -0.055 |
|  | Triglycerides | 0.101 (0.004) | 0.101 |
|  | HDL-cholesterol | 0.015 (0.005) | 0.015 |
|  | Waist circumference | 0.062 (0.007) | 0.062 |
|  | BMI | 0.148 (0.006) | 0.148 |
| **Type 2 diabetes status** | Age | 0.017 (0.001) | 0.153 |
|  | Sex | 0.220 (0.027) | 0.105 |
|  | Smoking status | 0.035 (0.014) | 0.035 |
|  | LLDS | -0.046 (0.012) | -0.045 |
|  | Income | -0.075 (0.014) | -0.074 |
|  | Triglycerides | 0.099 (0.014) | 0.096 |
|  | HDL-cholesterol | -0.138 (0.014) | -0.134 |
|  | Waist circumference | 0.221 (0.013) | 0.214 |
|  | Blood pressure | 0.056 (0.013) | 0.055 |
| **Covariances** |  | **Unstandardized estimates** | **Standardized estimates** |
| Triglycerides and HDL-cholesterol | | -0.397 (0.003) | -0.497 |
| Waist circumference and BMI | | 0.615 (0.003) | 0.821 |
| Income and education | | 0.396 (0.004) | 0.396 |
| Smoking status and risk drinking | | 0.317 (0.006) | 0.317 |
| LLDS and MVPA | | 0.122 (0.004) | 0.129 |
| **Variances^b^** |  | **Unstandardized estimates** | **Standardized estimates** |
| Income | |  | 0.967 |
| Education | |  | 0.947 |
| Smoking status | |  | 0.954 |
| LLDS | | 0.893 (0.005) | 0.894 |
| TV watching | | 0.867 (0.004) | 0.867 |
| MVPA | |  | 0.969 |
| Risk drinking | |  | 0.897 |
| Unhealthy sleep duration | |  | 0.950 |
| Triglycerides | | 0.859 (0.004) | 0.859 |
| HDL-cholesterol | | 0.741 (0.004) | 0.741 |
| Waist circumference | | 0.678 (0.003) | 0.678 |
| BMI | | 0.826 (0.004) | 0.826 |
| Blood pressure | | 0.791 (0.004) | 0.791 |
| Type 2 diabetes | |  | 0.826 |

^a^ Model fit indices: degree of freedom (*df*) 34, comparative fit index (CFI) 0.981, Tucker-Lewis index (TLI) 0.949, root mean square error of approximation (RMSEA) 0.032 (90%CI [0.031, 0.033]), and standardized root mean square residual (SRMR) 0.023. The model was estimated with the estimator weighted least squares with mean and variance adjusted (WLSMV), *n* = 68,649. Tests for significance: *p* value < 0.001 for all standardized path coefficients except for HDL-cholesterol to blood pressure (*p* value = 0.002) and smoking to incident type 2 diabetes (*p* value = 0.012). LLDS denotes Lifelines diet score and MVPA denotes non-occupational moderate-to-vigorous physical activity. Standardized values of LLDS, TV watching, waist circumference, BMI, and blood pressure (systolic blood pressure) were used for model estimation. Values of triglycerides and HDL-cholesterol were first log transformed and then standardized before model estimation. Sex denotes women compared with men.

^b^ Unstandardized variances (standard errors) of categorical variables were not available.

**Supplementary Table 4** Estimated associations between included risk factors and incident type 2 diabetes using logistic regression model (*n* = 68,649)^a^

| **Covariates** | **β-coefficients (95% CI)** | ***p* value** |
| --- | --- | --- |
| **Unmodifiable risk factors** |  |  |
| Women (versus men) | 0.355 (0.198, 0.512) | <0.001 |
| Age | 0.046 (0.039, 0.052) | <0.001 |
| **Modifiable risk factors** |  |  |
| **Socio-economic status** |  |  |
| Education (versus low) |  |  |
| Middle | -0.107 (-0.250, 0.036) | 0.143 |
| High | -0.140 (-0.322, 0.042) | 0.131 |
| Income (versus <1000 euro/month) |  |  |
| 1000-2000 euro/month | -0.219 (-0.509, 0.072) | 0.140 |
| 2000-3000 euro/month | -0.363 (-0.650, -0.077) | 0.013 |
| >3000 euro/month | -0.405 (-0.712, -0.098) | 0.010 |
| **Lifestyle behaviors** |  |  |
| Unhealthy sleep duration | 0.227 (-0.047, 0.501) | 0.105 |
| Risk drinking | 0.093 (-0.068, 0.254) | 0.256 |
| TV watching time | 0.071 (0.014, 0.129) | 0.015 |
| Lifelines diet score | -0.088 (-0.155, -0.021) | 0.010 |
| Smoking (versus never smoker) |  |  |
| Former smoker | 0.176 (0.037, 0.314) | 0.013 |
| Current smoker | 0.165 (-0.016, 0.346) | 0.074 |
| MVPA (versus lowest quintile)^b^ |  |  |
| 2^nd^ quintile | -0.200 (-0.405, 0.006) | 0.057 |
| 3^rd^ quintile | -0.177 (-0.376, 0.021) | 0.080 |
| 4^th^ quintile | -0.330 (-0.545, -0.115) | 0.003 |
| 5^th^ quintile | -0.129 (-0.336, 0.078) | 0.222 |
| **Clinical markers** |  |  |
| Triglycerides | 0.277 (0.207, 0.347) | <0.001 |
| HDL-cholesterol | -0.339 (-0.421, -0.258) | <0.001 |
| Waist circumference | 0.386 (0.271, 0.501) | <0.001 |
| BMI | 0.176 (0.077, 0.274) | <0.001 |
| Blood pressure^c^ | 0.156 (0.097, 0.215) | <0.001 |
| Constant | -6.738 (-7.238, -6.239) | <0.001 |

^a^ Standardized values of continuous variables (except for age) were used for risk estimation; triglycerides and HDL-cholesterol were first log transformed and then standardized.

^b^ MVPA denotes non-occupational moderate-to-vigorous physical activity.

^c^ Systolic blood pressure was used for risk estimation.

**Supplementary Table 5** Prevalence and incidence of type 2 diabetes in the Lifelines cohort and across several other countries/cohorts

(a) Prevalence

| **Countries** | **Prevalence, %** | **Region** | **World Bank income levels** |
| --- | --- | --- | --- |
| Lifelines cohort | 4.5 | Western Europe | High income |
| The Netherlands | 5.1 | Western Europe | High income |
| Brazil | 4.7 | South America | Upper-middle income |
| China | 6.0 | East Asia | Upper-middle income |
| Egypt | 3.3 | North Africa | Lower-middle income |
| India | 4.1 | South Asia | Lower-middle income |
| Japan | 5.5 | East Asia | High income |
| Nigeria | 1.0 | West Africa | Lower-middle income |
| Poland | 7.8 | East Europe | High income |
| Spain | 9.2 | Southern Europe | High income |
| Uganda | 1.6 | East Africa | Low income |
| United States | 10.0 | North America | High income |
|  | 7.9 |  | High income |
|  | 5.6 |  | Upper-middle income |
|  | 3.5 |  | Lower-middle income |
|  | 1.9 |  | Low income |

* For comparison of prevalence with other countries, we used the database - Global Health Data Exchange (GHDx) query tool (http://ghdx.healthdata.org/gbd-results-tool), established by Global Burden of Diseases, Injuries, and Risk Factors Study (GBD). We selected several countries that are representative of different socio-economic status and also with some focus on European countries. We used the data from year 2010 for checking prevalent type 2 diabetes, which is comparable to the data collection timeline of the Lifelines cohort study.

(b) Incidence

| **Cohorts** | **Incidence, %** | **Incidence rate per 1000 person-years** | **Person-years** | **Countries** |
| --- | --- | --- | --- | --- |
| Lifelines | 1.6 | 4.9 | 230,259 | The Netherlands |
| HELIUS-Dutch origin | - | 3.0 | - | The Netherlands |
| Rotterdam study | 9.5 | 11.9 | 54,024 | The Netherlands |
| Nurses’ Health | 7.1 | 3.4 | ~1.8 million | United States |
| MESA | 11.2 | 11.4 | 57,456 | United States |
| Whitehall II | 11.3 | 8.0 | 102,765 | United Kingdom |
| UK Biobank | 1.4 | 2.6 | 116,956 | United Kingdom |
| NutriNet-Santé | 0.8 | 1.4 | 582,252 | France |
| SUN | 0.9 | 0.8 | 215,149 | Spain |
| Swedish Military Conscription Registry | 2.2 | 0.9 | ~39.4 million | Sweden |
| ELSA-Brasil | 7.5 | 20.0 | 14,929 | Brazil |
| China Kadoorie Biobank | 3.2 | 3.3 | ~4.5 million | China |
| Tehran lipid and glucose study | 7.1 | 13.8 | 23,698 | Iran |

* For comparison of incidence with other countries, we searched through PubMed database and selected representative cohorts all over the world. We did not rely on National level data because of the substantial variations in follow-up time and methodological differences. For source references of these cohorts (doi number): HELIUS 10.1038/s41598-019-56596-4; Rotterdam study 10.1007/s10654-018-0414-8; Nurses’ Health 10.1093/jn/nxab195; MESA 10.1136/bmjdrc-2015-000185; Whitehall II 10.1136/bmj.e5452; UK Biobank 10.1016/j.clnu.2020.12.018; NutriNet-Santé 10.1001/jamainternmed.2019.5942; SUN 10.1016/j.clnu.2021.03.039; Swedish Military Conscription Registry 10.1007/s00125-015-3846-7; ELSA-Brasil 10.1016/j.diabres.2021.108747; China Kadoorie 10.1007/s00125-020-05091-x; Tehran lipid and glucose study 10.1016/j.jclinepi.2021.08.026

**Supplementary Table 6** Comparisons of baseline characteristics between included participants and those who lost to follow-up

|  | **With follow-up data** | **Lost to follow-up** |
| --- | --- | --- |
| Number of participants | 68,649 | 21,077 |
| Age, years | 49.7 (9.5) | 48.3 (9.8) |
| Female, % | 58.4 | 58.1 |
| Fasting glucose, mmol/L | 4.96 (0.51) | 4.97 (0.50) |
| HbA_1c_, % | 5.55 (0.30) | 5.54 (0.30) |
| Triglycerides, mmol/L | 1.19 (0.80) | 1.21 (0.88) |
| HDL-cholesterol, mmol/L | 1.53 (0.41) | 1.51 (0.41) |
| BMI, kg/m^2^ | 26.2 (4.0) | 26.5 (4.3) |
| Waist circumference, cm | 91.0 (11.7) | 91.4 (12.2) |
| Systolic blood pressure, mmHg | 126.4 (15.5) | 125.9 (15.7) |
| Never smoker, % | 42.4 | 44.6 |
| Lifelines diet score (no scale, max. 48) | 24.2 (5.9) | 23.3 (6.0) |
| Non-occupational MVPA, minutes/week^a^ | 190 (65, 370) | 180 (60, 360) |
| TV watching, hours/day | 2.5 (1.3) | 2.6 (1.4) |
| Alcohol intake, grams/day | 4.57 (0.89, 11.11) | 3.62 (0.72, 10.72) |
| Risk drinking (>15 grams/day), % | 16.7 | 16.7 |
| Unhealthy sleep duration, %^b^ | 2.97 | 4.13 |
| Low education, % | 31.2 | 36.3 |
| Low income (<1000euro/month), % | 3.0 | 4.3 |

^a^ MVPA denotes moderate-to-vigorous physical activity level.

^b^ Unhealthy sleep duration: <6 hours or >9 hours per day.

**Supplementary Table 7** STROBE statement - checklist of items that should be included in reports of cohort studies

|  | Item No | Recommendation |  | Page |  |
| --- | --- | --- | --- | --- | --- |
| **Title and abstract** | 1 | (*a*) Indicate the study’s design with a commonly used term in the title or the abstract |  | 2 |  |
|  |  | (*b*) Provide in the abstract an informative and balanced summary of what was done and what was found |  | 2 |  |
| Introduction | | |  |  |  |
| Background/rationale | 2 | Explain the scientific background and rationale for the investigation being reported |  | 5-6 |  |
| Objectives | 3 | State specific objectives, including any prespecified hypotheses |  | 6 |  |
| Methods | | |  |  |  |
| Study design | 4 | Present key elements of study design early in the paper |  | 7 |  |
| Setting | 5 | Describe the setting, locations, and relevant dates, including periods of recruitment, exposure, follow-up, and data collection |  | 7 |  |
| Participants | 6 | (*a*) Give the eligibility criteria, and the sources and methods of selection of participants. Describe methods of follow-up |  | 7-8 |  |
|  |  | (*b*) For matched studies, give matching criteria and number of exposed and unexposed |  | NA |  |
| Variables | 7 | Clearly define all outcomes, exposures, predictors, potential confounders, and effect modifiers. Give diagnostic criteria, if applicable |  | 8-12 |  |
| Data sources/ measurement | 8* | For each variable of interest, give sources of data and details of methods of assessment (measurement). Describe comparability of assessment methods if there is more than one group |  | *NA* |  |
| Bias | 9 | Describe any efforts to address potential sources of bias |  | 11-12 |  |
| Study size | 10 | Explain how the study size was arrived at |  | 7-8 |  |
| Quantitative variables | 11 | Explain how quantitative variables were handled in the analyses. If applicable, describe which groupings were chosen and why |  | 8-10 |  |
| Statistical methods | 12 | (*a*) Describe all statistical methods, including those used to control for confounding |  | 10-11 |  |
|  |  | (*b*) Describe any methods used to examine subgroups and interactions |  | 12 |  |
|  |  | (*c*) Explain how missing data were addressed |  | 11 |  |
|  |  | (*d*) If applicable, explain how loss to follow-up was addressed |  | 18 |  |
|  |  | (*e*) Describe any sensitivity analyses |  | 12 |  |
| Results | | |  |  |  |
| Participants | 13* | (a) Report numbers of individuals at each stage of study—eg numbers potentially eligible, examined for eligibility, confirmed eligible, included in the study, completing follow-up, and analysed |  | Suppl. Files |  |
|  |  | (b) Give reasons for non-participation at each stage |  | Suppl. Files |  |
|  |  | (c) Consider use of a flow diagram |  | Suppl. Files |  |
| Descriptive data | 14* | (a) Give characteristics of study participants (eg demographic, clinical, social) and information on exposures and potential confounders |  | 13 |  |
|  |  | (b) Indicate number of participants with missing data for each variable of interest |  | 24 |  |
|  |  | (c) Summarise follow-up time (eg, average and total amount) |  | 13 |  |
| Outcome data | 15* | Report numbers of outcome events or summary measures over time |  | 23 |  |
| Main results | 16 | (*a*) Give unadjusted estimates and, if applicable, confounder-adjusted estimates and their precision (eg, 95% confidence interval). Make clear which confounders were adjusted for and why they were included |  | Suppl. Files |  |
|  |  | (*b*) Report category boundaries when continuous variables were categorized |  | 8-9 |  |
|  |  | (*c*) If relevant, consider translating estimates of relative risk into absolute risk for a meaningful time period |  | NA |  |
| Other analyses | 17 | Report other analyses done—eg analyses of subgroups and interactions, and sensitivity analyses |  | 12 |  |
| Discussion | | |  |  |  |
| Key results | 18 | Summarize key results with reference to study objectives |  | 13-14 |  |
| Limitations | 19 | Discuss limitations of the study, taking into account sources of potential bias or imprecision. Discuss both direction and magnitude of any potential bias |  | 17-18 |  |
| Interpretation | 20 | Give a cautious overall interpretation of results considering objectives, limitations, multiplicity of analyses, results from similar studies, and other relevant evidence |  | 15-18 |  |
| Generalizability | 21 | Discuss the generalizability (external validity) of the study results |  | 18 |  |
| Other information | | |  |  |  |
| Funding | 22 | Give the source of funding and the role of the funders for the present study and, if applicable, for the original study on which the present article is based |  | 20 |  |

*Give information separately for exposed and unexposed groups.

**Note:** An Explanation and Elaboration article discusses each checklist item and gives methodological background and published examples of transparent reporting. Information on the STROBE Initiative is available at http://www.strobe-statement.org.
